# Remarks on Scarlet Fever as It Prevailed in Calloway County, Ky.

**Published:** 1840-08

**Authors:** W. Scott Lawrie

**Affiliations:** Kentucky


Art. 11.—*Remarks on Scarlet Fever, as it occurred in Calloway Co., Ky., in* 1838. By Dr. W. Scott Lawrie, of Ky.

In the following article I shall not attempt a labored dissertation on scarlet fever, but shall endeavor briefly to describe the symptoms and mode of treatment pursued during its prevalence in the county of Calloway, Kentucky, in the spring and early part of the summer of 1838. The winter had been unusually severe, and it was early in the spring that this formidable disease commenced its ravages. Its appearance created much alarm, the general impression that it was contagious pervading almost every family; and in some instances so great was the dread of spreading it, that the assistance so necessary among neighbors in times of sickness, was most inhumanly withheld, and a case or two occurred where barely sufficient force could be mustered to pay the last sad offices to the dead.

No disease with which I am conversant, is more appropriately named than "Scarlet Fever," a solitary symptom serving at once to develope its character. Its presence was variously intimated, at times being ushered in by rigors or slight chills, with redness of the eyes, pain in the head, heat, redness, and dryness of the skin, and difficulty of respiration; while again the *rash* itself, though not generally appearing until between the third and fifth day, was the first symptom manifested. In some other cases, soreness of throat was the symptom first complained of; and again, fever, redness of the eyes, the peculiar appearance and elevation of the papillæ of the tongue, resembling the seed of the strawberry, (a diagnostic in the cases of both black and white patients,) cough, wheezing, with a rattling of mucus in the throat, and redness and swelling of the fauces, were the symptoms which presented, in others.

These are the symptoms which usually came under my notice. The indications which they suggest are, first to abate the violence of the fever, and, secondly, to promote the cutaneous afflux or centrifugal tendency, scarlet fever, like all other eruptive diseases, being safest when it comes fully out upon the skin. Upon being called to a case in the commencement of an attack, and finding the usual symptoms attendant, particularly heat and redness of skin, with headache and difficult respiration, I endeavored to meet the first indication by abstracting blood freely from the arm, and in such a quantity as to satisfy me, that an impression had been made upon the symptoms manifested. Bloodletting as a remedy in scarlatina, I am aware, is not sanctioned by some of the profession, while by others it is as much extolled. Dr. McIntosh states, that for a good while he was opposed to it, but that upon finding so many of his cases snatched from an untimely grave, by *a spontaneous bleeding* from the nose, he determined upon a change of treatment; and at the time of his death he was an advocate for v. s. in this disease. So clear an indication of nature was not lost upon such a man as Dr. McIntosh.

The soreness and rattling in the throat, and the hurried and laborious respiration, would at once proclaim the propriety and necessity of an emetic, and, more particularly, should the cutaneous afflux be tardy in its appearance. Emetics, from their peculiar mode of operation, having a direct tendency to the skin, are very essential in the treatment of scarlatina, while cathartics, in their nature, are the reverse. An emetic in the onset, according to my experience, is the most effectual step towards cutting short the disease, and I am inclined to give the preference to tartar emetic over all the other articles of this class. In all the cases which have fallen under my care, I have used this remedy, and in only two have I seen it run off by the bowels, the objection urged against it, and in these, it failed to vomit altogether. In one instance only have I administered ipecac, and then, combined with calomel, it answered remarkably well. In the same family in which I gave it, I had six other patients all of whom took the antimonial and recovered.

To an abuse of any medicine I am opposed, and to none more than that of calomel as a purgative in scarlatina. Cathartics, I have stated, have no cutaneous tendency, and calomel least of all, being a permanent irritant of the mucous membrane of the alimentary canal, and reducing during the time of its operation the functions of the skin, which are in an inverse ratio to the peristaltic action.—In this disease the bowels are frequently sluggish and torpid, and that they should be opened, there can be no doubt; but that they should be moved by calomel is by no means certain. A majority of cases, in my opinion, will be as well, if not better managed without calomel, Epsom salts, and calcined magnesia, or rhubarb and magnesia, having with me done admirably well.

When the heat and dryness of skin continue after the stomach and bowels have been operated on, and the eruption is still tardy, or appears but partially, I have been much aided by the warm bath, either by immersion, or by pouring water from a vessel upon the body. Even sponging the body with warm water is of much benefit. The bath better than almost any other measure allays that restlessness so often attendant, and puts an end to the irritation of the skin, while at the same time it reduces the excessive temperature, promotes perspiration, and adds much to the comfort of the sufferer.

Cataplasms have also aided me much in the management of scarlet fever. A poultice of light-bread and milk, with the addition of cayenne pepper, has been usually employed. The temperature of the body is in many cases very unequally distributed, that of the abdomen being often preternatural, and in such instances a poultice covering the entire bowels will be found highly beneficial.

The testimony concerning vesication in this disease is conflicting. My own experience is favorable to it. In three or four cases, at least, I am confident that I obtained much advantage from the use of blisters; and I am not sure, that to them alone three of my patients are not indebted for their lives. In two of them, deglutition seemed impossible, and any liquid taken into the mouth was at once ejected through the nostrils. To the first I was called in much haste about 10 o'clock, P. M. The subject was a remarkably stout and healthy young woman, who had been for a few days convalescent, and had on that day been unfortunately exposed to a light shower of rain. I found her in extreme distress; her pulse full and bounding, skin hot and dry; much disturbance about the head, throat painful, and a good deal swollen. My efforts at v. s. failed entirely—the light was a bad one, and the patient being very fleshy, after two or three ineffectual attempts I abandoned it. Tartar emetic was forthwith administered, half a grain every fifteen minutes, but failed to induce emesis, the irritability of the bowels being such that it passed off that way. The effect of the watery stools thus procured was to exhaust the patient. The affection of the throat seemed rather to increase, and a poultice of light-bread and milk was prepared and applied as hot as could well be borne. From this some relief was obtained, and as she was kept in a state of nausea by a preparation of squill, and vin. antimon. combined with paregoric, some improvement was observable. The bowels had been acted upon by gentle cathartics, and I thought she was about to recover, when upon calling on the evening of the fourth day, I found her worse than ever. The poultices had been steadily continued, yet still the pain and swelling of the fauces, and the difficulty of deglutition were worse, and my apprehensions for her safety were much excited. A large blistering plaster was applied to the throat, and a strong decoction of cayenne pepper prepared, of which she was prevailed upon to drink freely, and under this plan of treatment, with due attention to the bowels, her health was restored. While in this, and some other cases, I am of opinion that vesication was productive of good, I am by no means prepared to say, that the practice should be generally adopted; on the contrary I look upon the cataplasms as safer, and as free from the objections so justly entertained against blisters. In my practice the epispastic has usually been employed after poultices and sinapisms had failed to secure the desired ends; and while I admit a doubt as to the general utility of vesication in this fever, I would unhesitatingly employ blisters, and risk gangrene, and the whole host of evils to which they are supposed to be accessary, before I would lose my patient without making the experiment.

Gargles I believe may be omitted. The cayenne pepper so prepared as to be comfortably swallowed, is a valuable adjuvant in the treatment of scarlatina, of which I have made much use, and which I can safely recommend. That the dryness of the skin should be obviated is very desirable, and this I have generally been able to effect by the use of the cayenne, and a preparation of vin. antimon., spt. nit. dulc., and paregoric, giving the pepper tea without limit, and, of the other, a teaspoonfull every hour, unless it acted too freely upon the stomach, in which case the time was prolonged.

Thus I have communicated the treatment pursued by me in the scarlet fever of Calloway county, and I may state, as the result, that out of some fifty cases thrown directly upon my hands, I lost but two, where the course of cure was begun by myself. Many received directions whose cases I never saw and were relieved; while others, laboring under the impression from having observed the great fatality of the disease elsewhere, that but little could be done by medical treatment, made no application for aid, until help was beyond reach, and thus cases were lost, two and three in a family, which by timely and judicious aid might have been saved.

The subjects of the fatal cases to which I have alluded were stout, robust children, one of them my own, whose sufferings were most intense, and upon whom no effort of mine made any impression. The brain became most deeply involved, and a heavy coma supervened, out of which I was unable to arouse her. The death of the other, from the peculiar formation of the child, I predicted while it was yet in health, should it become the subject of the disease. Every practitioner must have observed, that children of robust forms, short necks, and large heads, are the least likely to recover from scarlet fever.

The congestive form of scarlatina is not of very common occurrence, and out of the entire number of my cases, I am not sure that more than two were of that character. In one of these, the eruption never did fully appear. The child was sick some time before I saw it, was finally thrown upon the hands of another practitioner and lost, he with myself having failed in any manner to aid it. My experience in this form of the disease having been so limited I shall not pretend to speak of the best mode of treatment, but would simply remark, that while in inflammatory scarlatina I have dispensed with calomel, I should regard it as the most valuable remedy in cases of a congestive type.

In two instances I have met with a feature in this disease, that I have not seen described, which it may not be amiss to mention here. These patients were both convalescent, and one of them was able to leave the bed. They were taken with violent spasms of the forearm and hands; the contractions were very strong, and the suffering most excruciating and obstinate, not readily yielding to the means exhibited for its alleviation.

*June*, 1840.

